# Persistence of Anti-S1 IgG against SARS-CoV-2 Eight Months after the Booster Dose of Vaccine in Naive and Previously Infected Healthcare Workers

**DOI:** 10.3390/ijms241310713

**Published:** 2023-06-27

**Authors:** Sonia Algarate, Laura Serrano, Jessica Bueno, Beatriz Herrero-Cortina, Elena Alvarado, María T. González-Barriga, María Ducons, Jesica Montero-Marco, Sara Arnal, Beatriz Acha, María Riesgo, Ana Taboada, Pilar Sanz-Burillo, Cristina Yuste, Rafael Benito, on behalf of the RIPOVAC Study Group

**Affiliations:** 1 Hospital Clínico Universitario Lozano Blesa de Zaragoza, Avenida de San Juan Bosco, 15, 50009 Zaragoza, Spain; scajo@unizar.es (S.A.);; 2 Microbiology Department, University of Zaragoza, Calle de Pedro Cerbuna, 12, 50009 Zaragoza, Spain; 3 Instituto de Investigación Sanitaria de Aragón, Avenida de San Juan Bosco, 13, 50009 Zaragoza, Spain; 4 Nursing Department, University San Jorge, Autovía Mudéjar, km. 299, Villanueva de Gállego, 50830 Zaragoza, Spain

**Keywords:** SARS-CoV-2, booster vaccine, post-vaccine IgG antibody persistence

## Abstract

Our aim was to evaluate the immune response of healthcare workers included in the RIPOVAC study, after receiving a booster dose (third dose), in terms of intensity and persistence of induced antibodies. In the second phase of the RIPOVAC study, between December 2021 and January 2022, eight months after the second dose, 389 voluntary, immunocompetent, non-pregnant healthcare workers received a booster dose of SARS-CoV-2 vaccine, and a serum sample was obtained. Two groups of patients were established: with and without previous SARS-CoV-2 infection. In order to quantify anti-S1 IgG (AU/mL) we used CMIA (Abbott). All of the health workers were anti-S IgG positive 8 months after receiving the booster dose of the vaccine, with a mean of 17,040 AU/mL. In 53 patients without previous infection, antibody levels increased by a mean of 10,762 AU/mL. This figure is seven times higher than the one produced after the second dose (1506 AU/mL). The booster dose produces a robust elevation of the antibody level, which persists at 8 months, with levels significantly higher than those reached after the second dose, which allow one to predict a persistence of more than one year. The study demonstrates the efficacy of the booster dose of anti-SARS-CoV-2 vaccines.

## 1. Introduction

Since the start of the SARS-CoV-2 pandemic, various vaccines have been developed in order to control its spread [1]. In our setting, the most widely used vaccines have been mRNA-1273/Moderna and BNT162b2/Pfizer. These vaccines have been administered in two doses and the administration of a third booster dose has been recommended.

**Figure 1:**
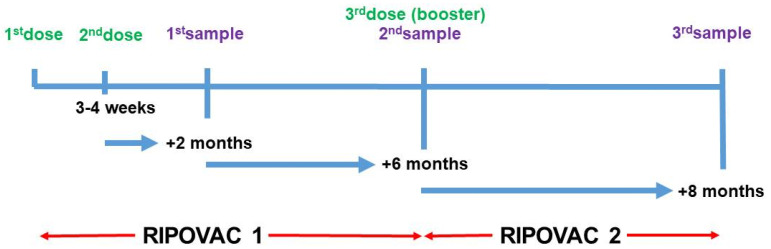
Schedule of RIPOVAC study. Data from RIPOVAC 1 in [2]. Third sample refers to samples analyzed in this study.

We undertook the RIPOVAC (Respuesta Immune Post-Vacuna COVID) study in 2021 (Figure 1). In its first phase (RIPOVAC 1), we studied the persistence of anti-SARS-CoV-2 post-vaccination antibodies at two and eight months after the second dose in a group of healthcare workers with and without previous infection [2].

We have now evaluated (RIPOVAC 2) the immune system response in relation to the presence, intensity, and persistence of SARS-CoV-2 anti-S1 antibodies 8 months after the administration of a booster dose in uninfected individuals and those previously infected.

## 2. Results

All of the health workers were anti-S IgG positive 8 months after receiving the booster dose of the vaccine (Table 1), with a mean of 17,040 AU/mL (95% CI, 7790 to 26,289) and a range between 483 and >40,000 AU/mL. This figure was 4 times higher than that obtained 8 months after the second dose of Moderna vaccine, which was 4297 AU/mL [2]. The mean number at 8 months after the third dose was 1.6 times higher [(17,962 AU/mL (95% CI, 9390 to 26,534))] in the patients infected at some point than in the patients without a history of infection [(11,190 AU/mL (95% CI, 795 to 21,585))] (Table 2). In both cases, the differences were statistically significant (*p* < 0.05).

**Figure 2:**
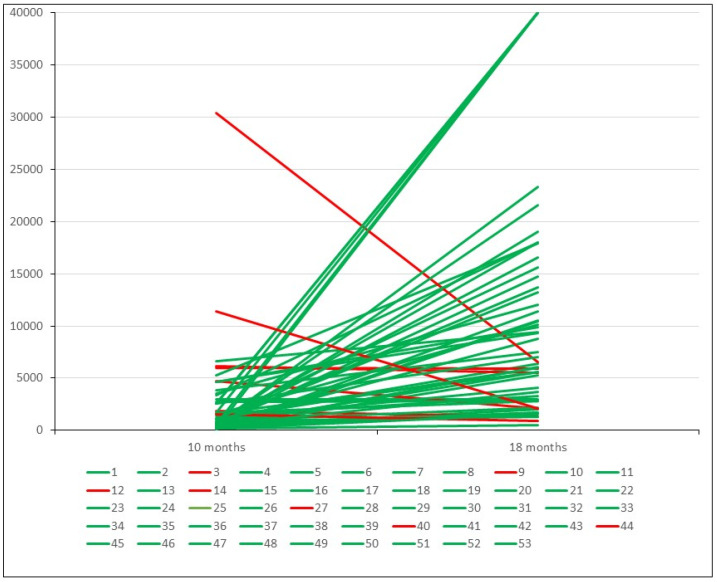
Antibody level variation eight months after third dose of vaccine in uninfected patients. Green lines indicate cases with elevation. Red lines indicate cases with a decrease.

Of the 53 uninfected healthcare workers who were studied eight months after the booster dose, the antibody levels increased in 46 (86.8%) and diminished in 7 (13.2%) (Figure 2). Nevertheless, the seven latter showed antibody levels between 920 and 6857 AU/mL (mean of 4104 AU/mL).

In these 46 patients, the antibody levels increased by a mean of 10,762 AU/mL at eight months after the booster dose. This figure is seven times higher than the mean value that these patients had 8 months after the second dose (1506 AU/mL). Fifty-one out of fifty-three cases (96.22%) showed antibody levels > 1300 AU/mL. 

In the uninfected patients who received a Moderna–Moderna–Moderna (MMM) regimen, after the third dose, a mean figure [(12,084 AU/mL (95% CI, 3965 to 20,203))] was detected that was higher than that of those who received the Pfizer–Pfizer–Moderna (PPM) regimen [(11,502 AU/mL (95% CI, −131 to 23,135))], but without statistically significant differences (*p* > 0.05) (Table 2). 

The lowest figures were detected in the patients who received a Pfizer–Pfizer–Pfizer (PPP) regimen, but the low number of patients in this group precludes the assessment of the significance of the difference.

**Figure 3:**
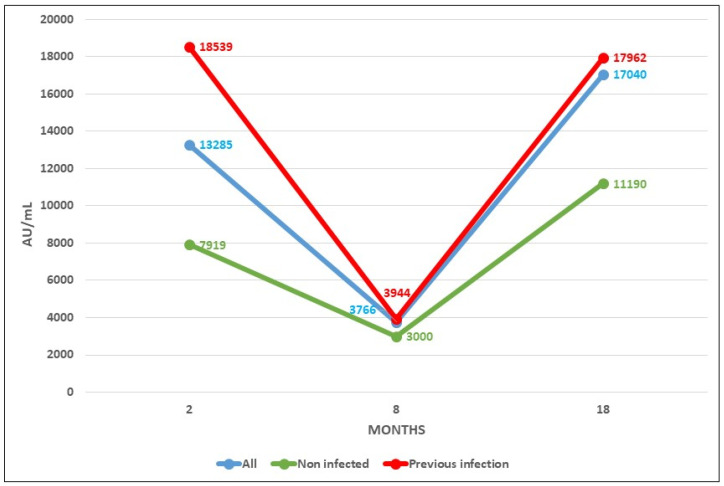
Antibody levels after COVID vaccine at 2, 8 (taken from [2]), and 18 months after vaccination.

Figure 3 shows the mean evolution of antibody level during both phases of the RIPOVAC study in the whole population studied and in naive patients. In both populations, an increased level of antibodies may be seen after the booster dose.

## 3. Discussion

The efficacy of the administration of two doses of vaccine for SARS-CoV-2 in the general population has been proven in terms of prevention of symptomatic infections [3,4] and, in particular, in terms of a decrease in the number of both symptomatic and asymptomatic infections in healthcare workers [5]. All of our infected healthcare workers were asymptomatic.

However, a decrease in antibody levels to 23.62% and 33.03% has been observed in those vaccinated with Moderna or Pfizer, respectively, 8 months after vaccination [2,6], although there are studies that show that the neutralizing capacity of antibodies can be maintained for up to 6–8 months [7,8].

In our current study, we have verified that the booster dose produces a robust elevation of the antibody level. These antibodies persist at 8 months, with levels significantly higher than those reached after the second dose, both in uninfected and previously infected healthcare workers. These data suggest a persistence of anti-S1 antibodies beyond 8 months. 

The increase in the concentration of antibodies after vaccination in people who had passed the infection has been verified in previous studies [9,10], and, as is shown in our study, this is true after the booster dose. 

As approximately 18 months have elapsed since the first dose of vaccine was administered to our healthcare workers and the Omicron (B.1.1.529) variant—which is characterized as being easily transmissible and able to escape the immune response [11]—was circulating in Spain, the number of uninfected healthcare workers initially included in our study has decreased considerably. However, the study of this group demonstrates the efficacy of the booster dose of the vaccine in terms of its ability to produce specific antibodies against SARS-CoV-2, also in uninfected healthcare workers, avoiding the bias of immunological memory conditioned by the stimulus of the immune system produced by previous infection. 

Longer studies are needed to verify the maximum persistence time of the antibodies after the booster dose and their efficacy against new variants that may be able to escape immunity, and to establish, if appropriate, new guidelines or reformulations of the vaccine.

## 4. Materials and Methods

We show data from the second phase of the RIPOVAC study, conducted at the Lozano Blesa University Clinical Hospital of Zaragoza, Spain, the reference center of Sector III of the Aragón Health Service (SALUD). The second phase of the RIPOVAC study (Figure 1) was carried out between December 2021 and January 2022, eight months after the second dose. At this time, 389 voluntary, immunocompetent, non-pregnant healthcare workers (55 men and 334 women, mean age of 47.87 years) (Table 3) received the booster dose of SARS-CoV-2 Moderna (377 cases, regardless of the brand received in the first two doses) or Pfizer (there were 12 cases that had received Pfizer in the first two doses) vaccine. Table 4 shows the characteristics of 53 healthcare uninfected workers according to the vaccination regimen. In the booster dose, only half of the usual dose of primary vaccination was administered, according to the recommendations of the COVID-19 Vaccination Technical Working Group of the Report on the Spanish Vaccination Program and Registry of 2 November 2021 [12]. 

At this time, a new serum sample was obtained [2]. The third serum sample was obtained 8 months after the booster dose. All serum samples were obtained via venepuncture. The healthcare workers were questioned about whether they had had compatible symptoms or whether they had been positive via a home antigen test for SARS-CoV-2. Their medical history was also reviewed to see whether they had a clinical history of SARS-CoV-2 infection (antigen, PCR, anti-N IgG, or IgM positive). 

People with a previous positive PCR for SARS-CoV-2 (Alinity m, Abbott; Viasure, Certest Biotec; or GeneXpert, Cepheid), with previous positive SARS-CoV-2 antigen (PanBio COVID-19 Ag, Abbott), or with presence of anti-N IgG (SARS-CoV-2 IgG, Abbott) or anti-SARS-CoV-2 IgM (SARS-CoV-2 IgM, Abbott) were considered as being previously infected.

The determination of quantitative anti-S1 IgG (AU/mL) was performed via CMIA (SARS-CoV-2 IgG II Quant) by blinded, trained laboratory staff, using the Alinity i platform (Abbott), following the manufacturer’s instructions. These reagent kits have a specificity of 100% and sensitivity of 98.77% at 15 days, according to the manufacturer, and are capable of detecting up to 40,000 AU/mL in undiluted serum.

The analysis was carried out based on the booster dose of the vaccine received and the existence or not of previous SARS-CoV-2 infection.

The protocol was approved by the Clinical Investigation Ethics Committee of Aragón (EPA 21/000) and the recruited healthcare workers gave their written informed consent, following the guidelines of the Declaration of Helsinki. 

Statistical analysis: Quantitative variables were described using the mean (standard deviation), and categorical variables were reported using frequencies. Differences in sociodemographic and serological data between the groups were tested using the independent Student *t*-test or the Mann–Whitney U and were reported as the mean difference (95% confidence interval, CI). Statistical significance was set at *p* < 0.05 for all calculations. Data analysis was performed using SPSS v.19 (IBM, Chicago, IL, USA).

## 5. Conclusions

The booster dose produces a robust elevation of the antibody level, which persists at 8 months, with levels significantly higher than those reached after the second dose. This fact seems to exclude the need for a second booster in uninfected healthcare workers. 

## Figures and Tables

**Table 1:** IgG anti-S1 response eight months after third dose of vaccine according to patient group.

	IgG ANTI-S1		
Patients	Positive	Mean AU/mL (SD)	Range
Total (389)	389 (100%)	17,040 (13,413.09)	483–>40,000
Uninfected (53)	53 (100%)	11,190 (11,013.24)	483–>40,000
Previously infected (336)	336 (100%)	17,962 (13,538.07)	969–>40,000

**Table 2:** IgG anti-S1 response eight months after third dose of vaccine in uninfected healthcare workers according to the brand of the three doses.

	IgG Anti-S1		
Patients	Dose 1st2nd3rd	Mean AU/mL (SD)	Range
15	MMM	12,084 (10,307.99)	919–>40,000
34	PPM	11,502 (11,804.34)	1291–>40,000
4	PPP	5180 (4528.49)	483–9933

M = Moderna; P = Pfizer.

**Table 3:** Characteristics of 389 healthcare workers with third dose of vaccine.

Sex (%) Mean Age (SD) [Range]	Previous Infection	
	Yes	No
55 male (14.14%) 46.34 (12.02) [22–65]	51 (13.11%)	4 (1.03%)
334 female (85.86%) 48.12 (11.16) [24–65]	285 (73.26%)	49 (12.60%)

**Table 4:** Characteristics of 53 uninfected healthcare workers according to the vaccination regimen.

Dose 1st2nd3rd	No. of Patients	Mean AU/mL (SD) [Range]	Sex (M/F)
MMM	15	52.66 (10.21) [29–60]	0/15
PPM	34	53.74 (7.79) [35–55]	3/31
PPP	4	61.5 (3.69) [55–64]	1/3

M = Moderna; P = Pfizer; M/F = male/female.

## Data Availability

All data are contained within the article; for further information, please contact the corresponding author.
